# Health care providers acceptance of default prescribing of TB preventive treatment for people living with HIV in Malawi: a qualitative study

**DOI:** 10.1186/s12913-023-10493-9

**Published:** 2024-01-04

**Authors:** L. M. De Groot, K. Shearer, C. Sambani, E. Kaonga, R. Nyirenda, K. Mbendera, J. E. Golub, C. J. Hoffmann, C. Mulder

**Affiliations:** 1 TB Elimination and Health System Innovations – KNCV Tuberculosis Foundation, The Hague, The Netherlands; 2 Center for Tuberculosis Research, John Hopkins University, Baltimore, MD USA; 3 Division of Infectious Diseases, School of Medicine, Johns Hopkins University, Baltimore, MD USA; 4 Department of Research, Ministry of Health, Lilongwe, Malawi; 5 KNCV Tuberculosis Foundation, Lilongwe, Malawi; 6 Department of HIV and AIDS, Ministry of Health, Lilongwe, Malawi; 7 National Tuberculosis and Leprosy Elimination Program, Ministry of Health, Lilongwe, Malawi; 8 Department of Epidemiology, Johns Hopkins Bloomberg School of Public Health, Baltimore, MD USA; 9 Department of Health, Behavior and Society, Johns Hopkins Bloomberg School of Public Health, Baltimore, MD USA; 10 Amsterdam Institute for Global Health and Development, Amsterdam University Medical Centres, Amsterdam, The Netherlands

**Keywords:** Tuberculosis, Preventive treatment, Choice architecture, People living with HIV, Acceptability, Health care providers

## Abstract

**Background:**

Tuberculosis (TB) preventive treatment (TPT) substantially reduces the risk of developing active TB for people living with HIV (PLHIV). We utilized a novel implementation strategy based on choice architecture (CAT) which makes TPT prescribing the default option. Through CAT, health care workers (HCWs) need to “opt-out” when choosing not to prescribe TPT to PLHIV. We assessed the prospective, concurrent, and retrospective acceptability of TPT prescribing among HCWs in Malawi who worked in clinics participating in a cluster randomized trial of the CAT intervention.

**Methods:**

28 in-depth semi-structured interviews were conducted with HCWs from control (standard prescribing approach) and intervention (CAT approach) clinics. The CAT approach was facilitated in intervention clinics using a default prescribing module built into the point-of-care HIV Electronic Medical Record (EMR) system. An interview guide for the qualitative CAT assessment was developed based on the theoretical framework of acceptability and on the normalization process theory. Thematic analysis was used to code the data, using NVivo 12 software.

**Results:**

We identified eight themes belonging to the three chronological constructs of acceptability. HCWs expressed no tension for changing the standard approach to TPT prescribing (prospective acceptability); however, those exposed to CAT described several advantages, including that it served as a reminder to prescribe TPT and routinized TPT prescribing (concurrent acceptability). Some felt that CAT may reduce HCW´s autonomy and might lead to inappropriate TPT prescribing (retrospective acceptability).

**Conclusions:**

The default prescribing module for TPT has now been incorporated into the point-of-care EMR system nationally in Malawi. This seems to fit the acceptability of the HCWs. Moving forward, it is important to train HCWs on how the EMR can be leveraged to determine who is eligible for TPT and who is not, while acknowledging the autonomy of HCWs.

**Supplementary Information:**

The online version contains supplementary material available at 10.1186/s12913-023-10493-9.

## Introduction

The African continent has the greatest burden of both tuberculosis (TB) and HIV [1]. Malawi, which is one of the World Health Organization’s (WHO) 30 high HIV-associated TB burden countries, has an estimated TB incidence rate of 141 per 100,000 and 54% of people diagnosed with TB are living with HIV [2, 3].

Antiretroviral therapy (ART) markedly reduces the probability of developing TB in PLHIV, up to 65% [4]. However, even at higher CD4 cell counts, PLHIV remain at elevated risk for developing TB [4, 5]. In addition to ART, TB preventive treatment (TPT) lowers the risk of developing TB among PLHIV and works both synergistically as well as independently with/from ART [4, 5]. Multiple systematic reviews have reported that TPT is effective, reducing TB incidence by up to 44% among PLHIV [6–8]. WHO recommends TPT for PLHIV: all PLHIV, regardless of age, should be screened for TB, and those who do not report any TB symptoms should receive TPT, while those who report symptoms should be given TPT once TB is excluded [9]. The WHO recently released consolidated guidelines that recommend the use of 3HP (weekly intake of isoniazid and rifapentine for 12 weeks) for PLHIV and (child) contacts of people with TB [9].

Despite the proven benefits of TPT, the WHO estimates that only 42% of PLHIV received it from 2005 to 2021 [1]. This “science-to-service gap”, the difference between what we know that works and what is provided to the person in practice, shows that clinical guidelines and policies often fail to achieve high levels of delivery of the intended clinical intervention [10]. The failure in TPT delivery is multi-factorial, including health care worker (HCW) related challenges with prescribing of TPT [11–15].

In 2017, the IMPAACT4TB^1^ project was initiated in Malawi; this five-year project focused on facilitating the introduction of shorter TPT regimens, including 3HP, in WHO recommended target groups [16]. Within the IMPAACT4TB project, a cluster randomized trial was conducted in three countries (Malawi, Mozambique, and Zimbabwe) which compared the choice architecture for TPT (CAT) strategy for TPT prescribing to the standard prescribing approach.

This study is based on the behavioral economics theory of choice architecture (from here referred to as CAT) which makes delivery of TPT the default option, such that HCWs need to opt-out when choosing not to prescribe TPT [17, 18]. This approach has been effective in multiple clinic implementation areas for other services [19–22]. We hypothesized that making TPT the default option normalizes TPT to be appropriate for all PLHIV (other than those with perceived exposure/risk). Furthermore, it helps to prevent HCWs from overlooking TPT at the initial encounter or after excluding TB disease (in case of TB symptoms) and shifts cognitive load to justifying not prescribing TPT rather than justifying TPT. We also hypothesized that through normalizing or routinizing TPT prescription to all (versus selecting for eligibility), the self-efficacy of HCWs regarding TPT prescribing may increase.

Successful implementation of an intervention depends, among other factors, on its acceptability to both intervention recipients and deliverers. If an intervention is considered acceptable, it is more likely to be delivered as intended, which has a positive impact on the overall effectiveness of the intervention [23]. To determine the acceptability of default prescribing of TPT to PLHIV, in-depth interviews were conducted with HCWs who worked in clinics participating in a cluster randomized trial of the CAT intervention; here, we present the results of this qualitative study.

## Methods

### Design

This is a multi-site qualitative study with a phenomenological character to assess the acceptability of the choice architecture for TPT (CAT) approach. We conducted in-depth, semi-structured interviews with clinic staff, of both intervention (CAT implementation) and control (standard implementation) clinics in Malawi. The CAT approach was facilitated in intervention clinics using a default prescribing module built into the point of care EMR system. During a person encounter, the HCW worked through several standard screens in both intervention and control clinics (i.e., TB symptom screening, contraindications, etc.). In intervention clinics, the EMR pre-determined TPT eligibility for the person and pre-selected TPT, if eligible. In control clinics, HCWs had to decide if the person was eligible and actively select to prescribe TPT.

### Setting

A total of 19 clinics (9 intervention and 10 control) using the point-of-care national HIV Electronic Medical Record system (EMR) in Mzimba North and Mulanje districts of Malawi participated in the CAT study from April 2021 to March 2022. According to Malawian guidelines; all children and adults living with HIV (regardless of CD4 cell count) should receive TPT, without the need for a test for TB infection [24].

### Participants

For each clinic, a list of all ART/TPT routine care providers was made and those with less than three months of experience at the study site were excluded. The list was stratified by clinic, sex, and job cadre. A purposive sampling approach was used to select HCWs for in-depth interviews to ensure balance and variety in participant characteristics. A total of 28 participants were recruited, fourteen HCWs per arm, who were interviewed between March and April 2022.

### Data collection

An interview guide (Supplement 1) for the interviews was developed, based on two frameworks: Theoretical Framework of Acceptability and Normalization Process Theory (Supplement 2). The Theoretical Framework of Acceptability developed by Sekhon et al. includes seven constructs of acceptability: attitude (how an individual feels about the intervention with confidentiality and receipt of care and medical procedures), burden (effort required for the intervention), ethics (concurrence with value system), coherence (how well the intervention is understood), opportunity costs, perceived effectiveness, and self-efficacy (confidence in performing or participating in the intervention) [23]. The Normalization Process Theory is a theory that describes new intervention implementation along lines of work coherence, cognitive participation, collective action, and reflexive monitoring [25] These two theories were chosen because they are complementary theories that help to explain implementation, especially implementation of a new approach, among service providers within a clinical setting.

Trained local researchers with experience in qualitative research conducted the face-to-face in-depth interviews at the clinics. The first interviews were transcribed by another local researcher for review and feedback. Subsequently, we slightly adjusted the interview guide by simplifying a few questions and removing some, but did not exclude the first interviews. All interviews were audio-recorded and transcribed verbatim (and translated if not in English).

### Data analysis

We used a thematic analysis approach, as it is useful for examining the perspectives of different research participants, highlighting similarities and differences, and generating unanticipated insights. This allowed us to identify, analyze, organize, describe, and report themes as found within the data [26]. Both deductive and inductive coding was used: deductive coding was guided by constructs from the Theoretical Framework of Acceptability and Normalization Process Theory and inductive coding allowed for unanticipated themes to arise from the data [23] (Supplement 3 shows the codebook). Two researchers (LMG and Lia Kerrigan [LK]) familiarized themselves with the data. They analysed the data with QSR Nvivo12 software, using the following roadmap:

1. *Open coding*: codes were applied to parts/sentences of the transcript. These codes were put under the constructs from the frameworks. LMG and LK double coded seven transcripts.
2. *Axial coding*: codes were discussed between LMG and LK and overarching themes were described. These themes showed how the framework constructs were reflected in this study. A coding scheme with themes and their definitions was built. This scheme was presented and discussed with CM, CJH, and KS; after which adjustments were made. Consequently, the seven double coded transcripts were coded again as well as an additional four transcripts (by LMG and LK), through which new themes arose. These were discussed and the research group agreed on a final coding scheme. LMG coded the remaining 17 transcripts.
3. *Selective coding*: associations and relations between themes were described, along the chronological design of the Theoretical Framework of Acceptability. This resulted in the development of summarizing theories, which were discussed with CM, CJH, and KS and were adjusted accordingly.

### Data reporting

The themes, as defined during axial coding, were categorized under the three chronological constructs of acceptability: prospective (prior to the CAT-intervention), concurrent (during CAT-intervention), and retrospective (after CAT study implementation). By organizing the results in such a way, the relationship between themes within one chronological construct can be clearly illustrated, as well as the relation between the different stages of acceptability. Also, it allowed us to use information given by HCWs from control clinics for prospective acceptability, and information given by HCWs from intervention clinics for prospective, concurrent, and retrospective acceptability. We used the COREQ (Consolidated criteria for Reporting Qualitative research) Checklist for presenting our study (Supplement 4).

## Results

Most study participants were professional nurses (43%) or medical assistants (29%) and had more than four years of work experience in the field of HIV/TB (68%) (Table 1).

**Table 1:** Study population characteristics

Sex n(%)	
Female	13(46)
Male	15(54)
Cadre n(%)	
Enrolled nurse	1(4)
Professional nurse	12(43)
Clinical officer	6(21)
Medical assistant	8(29)
Nurse midwife technician	1(4)
Years of experience in TB/HIV field n(%)	
< 1 year	1(4)
1–4 years	7(25)
> 4 years	19(68)
Unknown	1(4)
Age Median (IQR)	36(30; 44)

A total of eight themes belonging to the three chronological constructs were identified. Table 2 gives an overview of the different themes and their relation with the acceptability chronology. The summarizing theory below briefly explains the relationship between the themes and time constructs. Thereafter, all themes are explained individually.

### Summarizing theory

The prospective acceptability or value of making TPT prescribing the default option was quite low. All HCWs understood the importance of TPT prescribing (1), they perceived TPT prescribing as part of their job (2), and knew how to determine eligibility for TPT (3). Moreover, all HCWs experienced barriers in providing HIV/TB related care; resolving those issues seemed to have higher priority (4) than making TPT prescribing the default option. Yet, the concurrent acceptability was considered high; HCWs who experienced the CAT intervention were generally satisfied with the new approach and reported that it added value through reducing work and speeding up the prescription process (5). Although, a few HCWs expressed some criticism (6), which could partially be explained by the fact that not all HCWs seemed to fully understand the components of the CAT intervention (7). The retrospective acceptability was high as well; HCWs appreciated the CAT intervention, as they felt that TPT uptake had increased since the intervention (8).

**Table 2:** Overview of different themes and their relationship with chronological constructs

Chronological construct	Theme
Prospective acceptability (prior to CAT-intervention)	1. Importance of TPT is understood 2. Part of the job 3. Confidence in decision-making 4. Prevailing barriers
Concurrent acceptability (during CAT-intervention)	5. Perceived advantages of CAT: *lessens the work and/or it is faster, reminder, routine* 6. Perceived disadvantages of CAT: *CAT reduces critical thinking, concerned for over-prescribing* 7. Understanding of CAT procedure is minimal
Retrospective acceptability (post CAT-intervention)	8. TPT uptake has increased

### Prospective acceptability

#### 1. Importance of TPT is understood

Almost all HCWs explained that TPT decreases the risk of developing active TB for PLHIV. All HCWs were aware that they should prescribe TPT; half of them explicitly mentioned that they “don’t forget about TPT”. The two HCWs who did not find TPT helpful, reported not having the data on TB development, to decide whether TPT really decreases the risk of active TB. Most HCWs stressed that counseling PLHIV – to make sure they understand why they should take TPT medication – is of outmost importance, as well as asking for their consent to start medication. The majority of HCWs stated that PLHIV understand why they take TPT and therefore adhere to treatment.

*“Very good intervention, since it* (TPT) *has shown that most people who are HIV positive, they were dying of TB. So, now because of the TB preventive therapy that is being implemented, it means that a lot of people are being saved and then we might also have reduced numbers of people that can be diagnosed to have TB among people living with HIV”* – (Female, medical assistant, control arm, participant 12).

*“For sure yes, they* (PLHIV) *do understand because we explain why we are giving the TPT and usually we do explain in relation to HIV and TB. So when we explain, they understand and that’s why we don’t have… those that defaulted on TPT.”* – (Female, professional nurse, control arm, participant 11).

#### 2. Part of the job

When HCWs were asked whether TPT prescribing affects their workflow or adds to their workload, most HCWs said it did not. For them prescribing TPT is “*part of the job*” and HCWs explained that they incorporated TPT prescribing with ART prescribing. Moreover, the transition to 3HP (from 6H) made prescribing easier according to half of HCWs; e.g., fewer pills to prescribe and shorter treatment duration. Most HCWs estimated they needed 2–10 min for the TPT prescribing process and only a few HCWs thought it took more time.

*“It doesn’t take long because, each and every day those four cardinal signs, each and every person we are not giving TPT we also ask those four questions. So it’s just routine work we ask those questions. We ask are you pregnant? Are you on contraceptives? Those questions are not new. They are routine questions each and every day. So it doesn’t take time for TPT”* –.

(Female, professional nurse, control arm, participant 25)

Yet, HCWs did mention that the counseling – on HIV/TB in general, but also TPT specifically – at the start of treatment was time consuming. This was noted as a challenge on busy clinic days. Even though HCWs might find the counseling time consuming or experience it as a burden, they take time to give proper counseling because they are aware of the importance.

*“So yah, we have to see the process of disseminating information to someone is not an easy process, it’s not an easy task. So, you have, it involves giving and waiting for response and at the same time waiting for questions if any. So, I think, on TPT, mostly it becomes hard when you have a busy day”* – (Male, professional nurse, intervention arm, participant 2).

#### 3. Confidence in decision-making

All HCWs, from both intervention and control clinics, mentioned that they were confident in deciding who to prescribe TPT to and selecting the correct dose. All participants reported knowing how to determine TPT eligibility, and what the contraindications were including pregnancy and alcohol abuse. However, some of HCWs stated that there were discrepancies in the country’s guidelines versus job aids (posters) provided by the 3HP facilitation project regarding dose. In addition, HCWs mentioned that training and orientation helped to gain an understanding of when and how to prescribe, and almost all indicated that they consulted their colleagues when in doubt.

*“Cases where we are not sure of what to do, we consult our colleagues because we are aware that we are dealing with lives of people, hence we handle with care”* – (Male, nurse midwife technician, control arm, participant 16).

#### 4. Prevailing barriers

Even though all HCWs mentioned that TPT prescribing was easy, they did report barriers related to HIV/TB care: high pill burden, side effects, stigma (by PLHIV) and frequent stock-outs, job aids that don’t match guidelines, and duplicative prescribing of TPT (by HCWs). Also, technical problems were encountered; either related to electricity cuts or computer and/or EMR software related challenges. For example, at the time of the interviews, the EMR system did not include the fixed-dose combination for 3HP, which hampered prescribing and made monitoring adherence challenging.

*“It’s the pill burden because they are already taking ART some of them maybe they are already taking other non-communicable disease medications so for them to add with the TPT it was quite a challenge”* – (Female, clinical officer, control arm, participant 8).

*“If there is no power supply at the time when we want to assist clients, it cannot work because the EMR works when the power is on”* – (Female, medical assistant, intervention arm, participant 1).

### Concurrent acceptability

#### 5. Perceived advantages of CAT

##### Lessens the work and/or it is faster

Participants reported appreciating how the EMR guided them through steps of determining TPT eligibility. With the CAT approach TPT medication was automatically pre-ticked, whenever a person was eligible. According to HCWs, this lessened the workload and made TPT prescribing faster. HCWs experienced this as an advantage compared to the standard prescribing approach.

*“This system is just so fine. Because it lessens work for us as providers. As I have already said, if you have entered the right vitals, weight, and the like, whenever you have crossed on that to say this one is eligible for the 3HP. It will also go further up to an extent of giving how much drugs to be given to this patient”* – (Male, medical assistant, intervention arm, participant 19).

##### Reminder

More than half of the HCWs from intervention clinics perceived CAT as a valuable reminder to prescribe TPT. They explained that the fact that TPT medication is pre-ticked helped them in “not forgetting” that you should prescribe TPT to PLHIV.

*“It is good because it reminds us. Sometimes, it may happen that the provider has just forgotten to give the drugs but when those drugs appear on the EMR, it rings a reminder for you to give the drugs to the client or to tell the client about TPT”* – (Female, professional nurse, intervention arm, participant 22).

##### Routine

Choice architecture made TPT prescribing the default option, which made prescribing it more routine.

*“I feel like it’s good making the routine prescription for TPT because we cover a lot of clients who are eligible for getting this TPT”* – (Female, professional nurse, intervention arm, participant 28).

#### 6. Perceived disadvantages of CAT

##### CAT reduces critical thinking

Some HCWs felt that pre-ticking TPT in the EMR system may have reduced critical thinking. Following the guidance of the EMR was viewed by one participant as reducing HCW autonomy, to think for themselves, and potentially is overly directive. One HCW described it as the EMR prevents her to “think outside the box”.

*“For the EMR, what I liked about it is all the processes are actually fed up there, they’re in the EMR, so you just follow. You don’t have any other questions outside, just follow what is in the EMR. So that one is very easy. The only thing I don’t like of EMR is you are actually like one is getting directed, so this one doesn’t give much room to think outside the box, yeah, and the only concentrate on what is fed, what is actually in the EMR. Yeah, so that’s the only challenge”* – (Male, clinical officer, intervention arm, participant 24).

##### Concerned for over-prescribing

About half of the HCWs from intervention clinics expressed concerns with the system selecting persons for TPT. They mentioned that HCWs should always check whether a person is truly eligible for TPT. A few HCWs were afraid that making TPT prescribing the default option leads to over-prescribing of TPT. HCWs raised concerns that TPT could be prescribed to people who are not eligible for TPT. However, they did not give examples of prescribing to the wrong person, nor did they suggest that its something they had personally experienced.

*“Yeah, it’s not good enough, because it, it poses a threat somehow. It poses a threat in the sense that we will end up prescribing TPT to the wrong people. Wrong people in the sense that we do not exhaust much of the diagnostic approaches on TB and then at the end of the day, we end up giving TPT to everyone”* – (Male, clinical officer, intervention arm, participant 24).

#### 7. Understanding of CAT procedure is minimal

All HCWs working at intervention clinics were asked how CAT works. Most HCWs explained that it gives an opportunity to prescribe TPT with ART and repeated the importance of TPT for PLHIV. Next to this, some HCWs mentioned that it uses guiding questions to help HCWs decide whether persons are eligible – however, the EMR in control clinics also works with these guiding questions. Also, a few HCWs did not seem to understand immediately that their clinic was part of the CAT intervention until it was explained to them.

*“I may not be 100% correct in my explanation, but it probably it’s the, is the procedure which is put there to give TPT to those who are HIV positive, irrespective of whether got signs or not of the signs, as long as somebody is HIV positive, he has to get the TPT. That’s my understanding”* – (Male, clinical officer, intervention arm, participant 24).

### Retrospective acceptability

#### 8. TPT uptake has increased

The HCWs from intervention clinics were asked whether they thought TPT uptake changed after CAT implementation. Almost all HCWs thought that CAT increased the uptake of TPT and they saw this as a good thing. So even though they did not see the need in the beginning, because they already knew who to prescribe to, in hindsight, they did see an improvement.

*“I think maybe clients some clients maybe would have been missed out during the routine care but I think maybe with CAT we give to all eligible clients”* – (Female, professional nurse, intervention arm, participant 28).

## Discussion

In this study, HCWs showed high acceptability towards a choice architecture of default TPT prescribing for PLHIV. The HCWs who used CAT mentioned several advantages, including the reminder to prescribe TPT, a reduction of workload, and making prescribing TPT to PLHIV routine. Each of these reported benefits could, potentially, increase TPT prescribing. This is especially true if these advantages were significantly distinct from the alternative prescribing approach. It is also plausible, and a goal of the CAT implementation strategy, that through normalizing routine TPT prescribing clinician self-efficacy around TPT decision-making may have also increased, although this was not as clearly measured.

While the overall impression expressed by HCWs was positive, many participants reported that the prior approach to TPT prescribing was easy and system changes were not needed. Notably, TPT prescribing data for Malawi suggests prescribing below target goals suggesting barriers to prescribing do exist. Some participants suggested that health system related barriers beyond decision making challenges that served as impediments to TPT prescribing, for example software issues and medication stock-outs. In addition, some HCWs expressed concerns that the default approach could lead to overprescribing because they were unsure whether the EMR-system correctly selected persons for TPT and thought that HCWs may not appropriately note reasons for not prescribing. A similar concern was voiced by a few HCWs who were concerned about reduced autonomy with the CAT strategy. This highlights that training material regarding choice architecture needs to empower HCWs and amplify that default prescribing can complement daily work rather than replacing their professional integrity.

Choice architecture has a robust foundation outside of the field of health service delivery [18, 27]. One of the most notable success of a choice architecture approach is to substantially increase pension saving through making employee contribution the default [28]. Choice architecture has also had success in the healthcare to increase client or patient participation, for example with organ donation and with clinicians for cardiac rehabilitation referral after myocardial infarction and influenza vaccine prescribing, among other activities [18, 29, 30]. We are unaware of a similar example of deliberate choice architecture for TB prevention or HIV care overall. In all choice architecture approaches, the underlying principle is that actively selecting an option (the alternative to the default) takes more cognitive effort than potentially effortless passive acceptance of the default. By making the usually superior choice the default option, cognitive effort can be reserved for times when active decision making will be most valuable and the effect of cognitive fatigue on decision making will have a less negative impact.

Responses from participants who worked at a CAT clinic suggest that underlying expectations of choice architecture were achieved – included reduced decision making and potentially greater prescription of TPT. The responses also raised important implementation considerations and the likelihood that the CAT strategy was not optimally implanted in all clinics. The need for thoughtful choice architecture strategy design, including effective engagement of the using community (e.g. HCWs), has been previously highlighted [29]. Some design limitations were raised by interview participants, some of which we sought to address during strategy implementation.

A major strength of our study was that half of the study participants had worked with the CAT intervention under close to programmatic conditions which reflected real practice with its associated challenges. Despite these challenges, the intervention was highly accepted. Another strength was the frequency and intensity of code and theme discussion. On a biweekly basis, code, coding tree, themes, and summarizing theories were discussed within the research team. This led to in-depth understanding of the data HCWs provided to us. To facilitate this, multiple interviews were double coded.

This study has several limitations. Just before study implementation began, the Department of HIV/AIDS from Malawi adopted the default TPT prescribing module nationally for PLHIV in clinics with the point-of-care EMR system. As a result, the default prescribing module had to be switched off in the control clinics to conduct the study. This may have led to confusion and contamination of control clinic HCWs as the default prescribing module was in use for two months prior to study implementation. Likewise, we also speculate that as default prescribing had been adopted as part of the national program, HCWs who transferred into a CAT intervention clinic from a non-CAT study site may have understood the default prescribing approach to be standard, as opposed to a study-specific intervention. As a result, much of the insights provided were about the EMR in general. Hence, some perspectives as presented under concurrent acceptability could have been interchangeably applicable to HCWs from control clinics, although we focused on using CAT specific findings as much as possible. We also observed some language barriers as all interviews, except three, were conducted in English. While this facilitated the process of data collection and analyses, it may have affected the richness of data as we observed that some questions were just partly understood.

## Conclusions

Default prescribing of TPT has become standard of care for PLHIV receiving care at clinics with the point-of-care HIV EMR in Malawi. This seems to fit the acceptability of HCWs working in HIV/TB care. Notably, HCW using the CAT system need to be engaged in the design and implementation process to avoid voiced concerns – such as overprescribing of TPT or lost autonomy – from undermining the expected increased prescribing of TPT.

### Electronic supplementary material

Below is the link to the electronic supplementary material.

Supplementary Material 1

Supplementary Material 2

Supplementary Material 3

Supplementary Material 4

## Data Availability

The datasets used and/or analyzed during the current study are available from the corresponding author on reasonable request.

## Footnotes

1 IMPAACT4TB: Increasing Market and Public health outcomes through scaling up Affordable Access models of short Course preventive therapy for TB - IMPAACT4TB - IMPAACT4TB.

## Abbreviations

3HP: Three months weekly dose isoniazid and rifapentine
ART: Antiretroviral therapy
CAT: Choice architecture
EMR: Electronic medical record
HCW: Health care worker
PLHIV: People living with HIV
TB: Tuberculosis
TPT: Tuberculosis preventive treatment
WHO: World Health Organization

## References

[1] WHO. Global Tuberculosis report 2022. Geneva; 2022.

[2] WHO. Global Tuberculosis report 2021. Geneva; 2021.

[3] Bank TW. Incidence of tuberculosis (per 100,000 people) - Malawi 2020 [Available from: https://data.worldbank.org/indicator/SH.TBS.INCD?end=2020&locations=MW&most_recent_value_desc=true&start=2020&view=bar.

[4] Pathmanathan I, Ahmedov S, Pevzner E, Anyalechi G, Modi S, Kirking H (2018). TB preventive therapy for people living with HIV: key considerations for scale-up in resource-limited settings. Int J Tuberc Lung Dis.

[5] Hamada Y, Getahun H, Tadesse BT, Ford N (2021). HIV-associated Tuberculosis. Int J STD AIDS.

[6] Ayele HT, Mourik MS, Debray TP, Bonten MJ (2015). Isoniazid Prophylactic Therapy for the Prevention of Tuberculosis in HIV infected adults: a systematic review and Meta-analysis of Randomized trials. PLoS ONE.

[7] Uppal A, Rahman S, Campbell JR, Oxlade O, Menzies D (2021). Economic and modeling evidence for Tuberculosis preventive therapy among people living with HIV: a systematic review and meta-analysis. PLoS Med.

[8] Briggs MA, Emerson C, Modi S, Taylor NK, Date A (2015). Use of isoniazid preventive therapy for Tuberculosis prophylaxis among people living with HIV/AIDS: a review of the literature. J Acquir Immune Defic Syndr.

[9] WHO. WHO operational handbook on tuberculosis (Module 1 - Prevention): Tuberculosis preventive treatment. Geneva; 2020.

[10] Dissemination and Implementation Research in Health. In: Colditz GA, Proctor EK, editors. Translating Science to Practice. Brownson RC. Oxford University Press; 2012. 24 May 2012.

[11] Teklay G, Teklu T, Legesse B, Tedla K, Klinkenberg E (2016). Barriers in the implementation of isoniazid preventive therapy for people living with HIV in Northern Ethiopia: a mixed quantitative and qualitative study. BMC Public Health.

[12] Mindachew M, Deribew A, Tessema F, Biadgilign S (2011). Predictors of adherence to isoniazid preventive therapy among HIV positive adults in Addis Ababa, Ethiopia. BMC Public Health.

[13] Wambiya EOA, Atela M, Eboreime E, Ibisomi L (2018). Factors affecting the acceptability of isoniazid preventive therapy among healthcare providers in selected HIV clinics in Nairobi County, Kenya: a qualitative study. BMJ Open.

[14] Lai J, Dememew Z, Jerene D, Abashawl A, Feleke B, Teklu AM (2019). Provider barriers to the uptake of isoniazid preventive therapy among people living with HIV in Ethiopia. Int J Tuberc Lung Dis.

[15] Lester R, Hamilton R, Charalambous S, Dwadwa T, Chandler C, Churchyard GJ (2010). Barriers to implementation of isoniazid preventive therapy in HIV clinics: a qualitative study. Aids.

[16] Institute A. Who we are 2018 [Available from: https://www.impaact4tb.org/who-we-are/.

[17] Johnson EJ, Shu SB, Dellaert BGC, Fox C, Goldstein DG, Häubl G (2012). Beyond nudges: tools of a choice architecture. Mark Letters: J Res Mark.

[18] Johnson EJ, Goldstein D (2003). Medicine. Do defaults save lives?. Science.

[19] Shaikh RA, Simonsen KA, O’Keefe A, Earley M, Foxall M, Islam KM (2015). Comparison of Opt-In Versus Opt-Out Testing for sexually transmitted Infections among inmates in a County Jail. J Correct Health Care.

[20] Abadie A, Gay S (2006). The impact of presumed consent legislation on cadaveric organ donation: a cross-country study. J Health Econ.

[21] Hayes R, Moulton LH. Cluster randomized trials. 2nd ed. Chapman and Hall/CRC Press; 2017.

[22] Danel C, Moh R, Gabillard D, Badje A, Le Carrou J, Ouassa T (2015). A trial of early antiretrovirals and Isoniazid Preventive Therapy in Africa. N Engl J Med.

[23] Sekhon M, Cartwright M, Francis JJ (2017). Acceptability of healthcare interventions: an overview of reviews and development of a theoretical framework. BMC Health Serv Res.

[24] Health Mo. Clinical management of HIV in children and adults – 2019 Policy updates (addendum to the 4th edition of the Malawi integrated guidelines and standard operating procedures for clinical HIV services. 2019.

[25] May CR, Mair F, Finch T, MacFarlane A, Dowrick C, Treweek S (2009). Development of a theory of implementation and integration: normalization process theory. Implement Sci.

[26] Nowell LS, Norris JM, White DE, Moules NJ (2017). Thematic analysis: striving to meet the trustworthiness Criteria. Int J Qualitative Methods.

[27] Kim RH, Day SC, Small DS, Snider CK, Rareshide CAL, Patel MS (2018). Variations in Influenza Vaccination by Clinic Appointment Time and an active choice intervention in the Electronic Health Record to increase Influenza Vaccination. JAMA Netw Open.

[28] Madrian BC, Shea DF (2001). The power of suggestion: inertia in 401(k) participation and savings behavior. Quart J Econ.

[29] Patel MS, Volpp KG, Asch DA (2018). Nudge units to improve the delivery of Health Care. N Engl J Med.

[30] Patel MS, Volpp KG, Small DS, Wynne C, Zhu J, Yang L (2017). Using active choice within the Electronic Health Record to increase Influenza Vaccination Rates. J Gen Intern Med.

