# Supplementary material for: Health care providers acceptance of default prescribing of TB preventive treatment for people living with HIV in Malawi: a qualitative study
Source: BMC Health Serv Res. 2024 Jan 4;24:15. doi: 10.1186/s12913-023-10493-9 (PMC10768226; doi:10.1186/s12913-023-10493-9)
Supplement: Supplementary file 1 — Supplementary Material 1 [file 12913_2023_10493_MOESM1_ESM.docx]

**Supplement 1 – Interview guide**

**Choice Architecture Based TB Preventive Therapy Prescribing: IMPAACT4TB Implementation Research**

**Short Title: “TPT Prescribing”**

**INTERVIEW GUIDE: Health Care Workers**

**PART A: General information:**

Thank you for taking the time to talk with me. I want to learn more about your experiences with TB preventive care for patients. The focus of our discussion will be on TPT prescribing. We are also interested in overall why and when you consider providing medication or guidance to a patient to prevent illness versus treating an illness. We will also discuss 3HP prescribing and the approach used at your clinic. Your participation in this conversation is completely voluntary, and may be stopped at any time. The information you provide will remain completely confidential and will be helpful to improve future TPT prescribing. Do you have any questions before we start?

For the purpose of this recording, do you agree to take part in this interview?

***Please tick an appropriate answer or fill the blanks accordingly***

1. Date:
2. Participant ID number:

| - MALAWI | - MOZAMBIQUE | - ZIMBABWE |
| --- | --- | --- |

1. Participant country:
2. Participant Clinic: _______________
3. Sex: Male [ ] Female [ ]
4. Age _____________ years
5. Job category

General practitioner / doctor ( )

Clinical officer ( )

Professional Nurse ( )

Enrolled Nurse ( )

Other (specify)___________________________

1. Years of experience in the HIV/TB field:

(a) Less than 1 year

(b) 1 - 4 years

(c) More than 4 years

**Part B: Interview**

General Clinic background

- Can you tell me about your role in the clinic?
  - How long have you been working here?
- Can you describe your role in providing care for people with HIV (PLHIV)?
- Do you prescribe ART and other meds for PLHIV, dispense, or recommend to others to prescribe?
- Do you provide adherence counseling or monitoring for PLHIV?
- How long have you been prescribing ART?
- How long have you been providing adherence support for PLHIV?
- When you see a patient who is just starting ART, what is going through your mind as the most important things that you need to get done for this patient?
  - What questions do you ask a patient?
  - What are common questions that patients ask you about their HIV and/or TB care?
  - What kinds of issues are patients concerned or worried about when initiating on ART(i.e. side effects of ART, disclosure to partners, opportunistic infections or TB, etc)?
- In your opinion, what are the most important steps to take when initiating a patient on ART? Please describe these steps.
- When you see a patient who is coming for ART continuation, what are the steps you go through in the visit?

Now we are going to talk about TPT prescribing. When I say TPT, I mean all TB preventive therapy – so both 3HP and IPT.

**Intervention coherence**

- Tell me about the process of prescribing TPT
- Who is responsible for prescribing TPT?
- How do you decide who to give TPT to?
- When do you consider prescribing TPT?
  - When someone is diagnosed with HIV?
  - When a patient first starts ART?
  - Or only after they have been on ART for some time?
- What do you do if someone has TB type symptoms?
  - What happens if you do a TB test and the test is negative?
  - Is there a reminder to think about TPT? Describe how this works.
- How do you usually determine if someone has been on TPT already or recently been on anti-TB treatment?
- Where do you record the TPT prescription?
- Is there anything you save for the next visit if the patient has lots of questions or for any other reason the consultation is taking a long time?
  - How do you decide what to save for the next visit or prioritize for the current visit?
- Has the process of prescribing TPT changed in the last few years? If so, explain how. Has this made it easier or harder to prescribe TPT?

**Affective attitude**

- What do you think about TPT as an intervention for TB prevention in PLHIV?
  - Have you had any positive experiences with TPT prescribing? Tell me about it:
  - Have you had any negative experiences with TPT prescribing (e.g. side effects, active TB, default)? Tell me about it:
- What are the challenges you face in TPT prescribing? Explain further.
  - Are there any other challenges? If yes, please describe those.
- Is there anything you would like to change regarding the way decisions are made regarding to whom to prescribe TPT?
- How do your patients respond to TPT?
- Do they understand why it is being prescribed?
- Do you think patients understand the value of TPT?
- To what extent do you think they adhere to TPT?
- What patient level factors (e.g. pill burden, stigma) affect your delivery of TPT?

**Ethicality**

- Do you think providers should prescribe TPT to most or all PLHIV? Why or why not?
  - What are your thoughts on whether TPT is good (or bad) for patients?
  - What concerns do patients have regarding taking TPT?
  - Tell me about TB stigma and how that affects you talking about TPT or patients accepting TPT.

**Self-efficacy**

- How confident are you in prescribing TPT?
  - Are you always sure that you are making the right decision about whether or not to give TPT?
    - Can you describe that further?
  - Tell me about a time when you weren’t sure about prescribing TPT to a patient.
    - What did you do?
    - How did the patient respond?
    - Who did you go to for information and/or advice?
    - What was the outcome?
- What factors do you consider when thinking about whether to prescribe TPT to a patient?

B**urden**

- Please explain how TPT prescribing fits into your daily workflow
- How about evaluating for TPT?
- How do you use laboratory results, notes in files, medical history or other sources of information in deciding whether or not to prescribe TPT?
- How long do you think it usually takes to assess whether TPT is appropriate?
- How does prescribing TPT to a patient affect your work load for the day?

**Opportunity Costs**

- Do you feel that TPT prescribing limits the time available to address your patient’s other concerns and conduct other duties? If so, tell me about that
- How is the TPT prescribing process different than for other diseases (e.g. TB treatment)?
  - Are there more things that you have to take into consideration? If yes, explain.

**Perceived Effectiveness**

- Do you have any suggestions for how to increase TPT prescribing for PLHIV?

The following is only for intervention clinics. If a control clinic, skip to the end.

Intervention (only for intervention clinics)

- Did you participate in the CAT study (using the default prescribing module in the EMR, pre-printed stamp or sticker, or TPT ticket system)? *(If participant says no, ask them if they were aware of the study?)*

**Intervention coherence**

- Describe the CAT approach to me as though you were explaining it to a co-worker who hasn’t heard of it before.

**Affective Attitude**

- How do you feel about the CAT approach to TPT prescribing?
  - Overall, describe whether you feel that making prescribing of TPT more routine is bad or good.

**Burden**

- How did the CAT approach affect your workflow?
- How did using the CAT approach affect your workload compared to routine TPT prescribing?
- How easy or hard was it to use the CAT approach for TPT prescribing?
  - Describe what made it easy or hard.
  - Tell me about the sticker/stamp/ticket/EMR that you used as part of the CAT approach. What did you like about it? What did you not like about it?
- Did the CAT approach help you at all? (Explain)
- What would you change about the CAT approach to TPT prescribing? Explain

**Ethicality**

- Would you recommend the CAT approach to TPT prescribing to a colleague? Why or why not?
  - Describe how the CAT approach affects patient care, in good ways and bad ways.

**Opportunity Costs**

- How did the CAT approach affect time you spent on other activities with a patient?
  - Did you have to give up other aspects of patient care to be able to use the CAT approach? Tell me about that.

**Perceived Effectiveness**

- In your opinion, how did the CAT approach affect TPT prescribing in your clinic?
  - Do you think TPT prescribing increased or decreased compared to routine care? How so?
- Tell me about any concerns you have about the CAT approach.
  - Do you think any patients received TPT through the CAT approach that shouldn’t have?
    - Tell me about those types of patients
  - What issues did patients report that were prescribed TPT under the CAT approach?
    - Were there any side effects? How did you respond if a patient reported a side effect?
- Tell me about how you assess TPT adherence and completion. Is this different under CAT than under routine TPT prescribing?
- Do you have any other concerns?

Thank you again for your time - your feedback has been very helpful. Before we close the interview, do you have any additional questions or comments regarding the CAT study?

The following is only for intervention clinics. If a control clinic, end the interview

**Part C. Acceptability of the Choice Architecture Intervention**

***Note to the interviewer, please review the definition of choice architecture with the participant****, i.e. that TPT should be prescribed with any ART initiation and any ART re-prescribing if TPT has not been previously prescribed. This approach of simultaneous prescribing is facilitated through the use of an EMR modification, ink stamp or pre-printed sticker, or ticket (dependent on country) for quick entry of the ART prescription* *along with TPT. We refer to this approach as the ‘choice architecture approach.’*

Complete all statements as honestly as possible. For each statement, indicate whether you agree with the statement, are neutral (neither agree or disagree), or disagree with the statement by making a “TICK” in one of the boxes [the choice that best represents your opinion/your choice]. **Be as honest as possible.**

|  | Question | Likert Scale |
| --- | --- | --- |
| 1 | Overall, I feel that the choice architecture approach is useful for TPT prescribing. | Agree  Neutral  Disagree |
| 2 | Overall, the choice architecture approach decreased my workload compared to routine TPT prescribing. | Agree  Neutral  Disagree |
| 3 | Overall, it took less effort (less thinking) to prescribe TPT using the choice architecture approach compared to routine prescribing. | Agree  Neutral  Disagree |
| 4 | I feel that the choice architecture approach is the right way to prescribe TPT. | Agree  Neutral  Disagree |
| 5 | I fully understand the choice architecture intervention and can explain it to a co-worker. | Agree  Neutral  Disagree |
| 6 | I feel that I had to give up other aspects of patient care or other clinic duties to use the choice architecture approach. | Agree  Neutral  Disagree |
| 7 | Regarding TB prevention, I feel that the choice architecture approach allowed me to provide better care for my patients. | Agree  Neutral  Disagree |
| 8 | In terms of care *unrelated* to TB prevention, I feel that the choice architecture approach allowed me to provide better care for my patients. | Agree  Neutral  Disagree |
| 9 | I think that the choice architecture approach increased TPT prescribing. | Agree  Neutral  Disagree |
| 10 | I am confident in prescribing TPT using the choice architecture approach. | Agree  Neutral  Disagree |
