# Supplementary figures and images for: Health care providers acceptance of default prescribing of TB preventive treatment for people living with HIV in Malawi: a qualitative study
Source: BMC Health Serv Res. 2024 Jan 4;24:15. doi: 10.1186/s12913-023-10493-9 (PMC10768226; doi:10.1186/s12913-023-10493-9)

**Supplement 2 – Theoretical framework of acceptability**

**
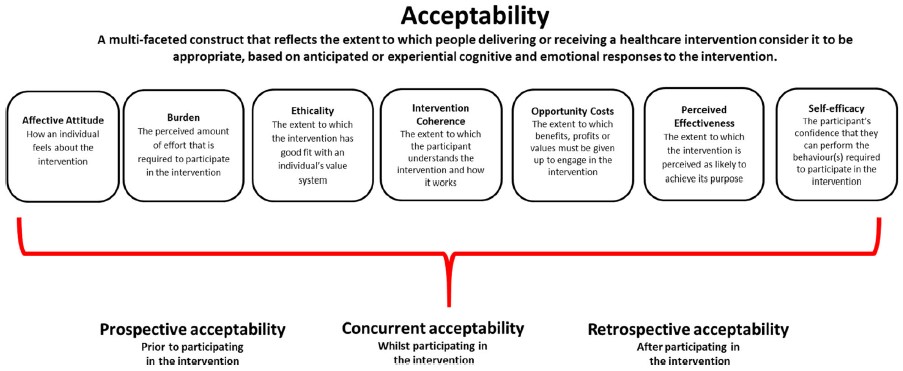
**

Supplement: Supplementary file 2 — Supplementary Material 2 [file 12913_2023_10493_MOESM2_ESM.docx]
