# Supplementary material for: Health care providers acceptance of default prescribing of TB preventive treatment for people living with HIV in Malawi: a qualitative study
Source: BMC Health Serv Res. 2024 Jan 4;24:15. doi: 10.1186/s12913-023-10493-9 (PMC10768226; doi:10.1186/s12913-023-10493-9)
Supplement: Supplementary file 3 — Supplementary Material 3 [file 12913_2023_10493_MOESM3_ESM.docx]

**Supplement 3 - Codebook**

| **Node** | **Child node** | **Description** |
| --- | --- | --- |
| *Barriers regarding TPT* | High pill burden | Most clients experience trouble with pill burden. Both for TPT alone, but also in combination with ART |
|  | Side effects | Refers to experienced side effects |
|  | Stigma (by PLHIV) | Clients experience stigma when taking TPT. e.g. others might think they have TB and take TB medication instead of preventive medication |
|  | Frequent stock-outs | Whenever health care facilities do not have enough medication in stock to prescribe to their clients. |
|  | Job aids don’t match guidelines | Providers indicated that TPT guidelines and educational materials were misaligned in terms of eligibility and technical guidance. |
|  | Duplicative TPT prescription | When HCWs (by accident) prescribe TPT to people more than once. |
|  | Electricity cuts | Refers to quotes on electricity cuts in clinics, making work more difficult |
|  | CAT leads to double admin | Record TPT prescription in EMR, health passport, mastercard, etc. |
|  | Clients concerns about TPT | Most clients have concerns about TPT – the food they must take with medication, stigma, pill burden, side effects, remembrance TPT medication day, and disease course. Those concerns emerged before someone starts on TPT. |
|  | Technical problems | All problems faced regarding TPT prescription/monitoring and/or medication supply that is digital/technical related |
|  | Understaffed and undertrained | Whenever colleagues of TPT providers are undertrained or the clinic they're working in is understaffed |
| *HCWs are confident to prescribe TPT* | Confident to prescribe TPT | HCWs feel confident to prescribe TPT |
|  | Work in a team | HCWs mention having colleagues they could ask for help. |
|  | Training | Follow training on how to prescribe TPT and/or use CAT. |
| *Importance of TPT is understood* | We don't forget about TPT | HCW mention TPT as one of the first things they think about when they meet a person newly diagnosed with HIV. |
|  | Determine eligible persons | The first step in TPT prescribing is deciding whether a person is eligible for TPT. |
|  | Build a relationship | For (in-depth) counseling it is important to build a relationship between HCW and client. |
|  | Take time for clients | It is important that HCWs take time for their clients to make sure they understand everything (do not leave them with unanswered questions) |
|  | Client has veto | It is always up to the client to decide whether he/she wants to take TPT. This is related to counseling because clients need proper counseling to make this decision. |
| *Prescribing TPT is easy* | Part of the job | Prescription of TPT is part of the job, and therefore not difficult, because “we do it every day” |
|  | No effect on workload | TPT is prescribed daily, and (therefore) HCWs do not experience TPT-prescription as a burden. Even though, they also have said that extra knowledge is needed to prescribe TPT. |
| *Advantages CAT* | Reminder | CAT serves as a reminder for TPT |
|  | Routine | The architecture makes TPT prescription the default option, TPT prescription now goes more automated which made it routine work |
|  | Prevents missing eligible people | CAT prevents HCWs from missing TPT eligible persons |
| *TPT uptake increased* | TPT uptake increased | CAT increases TPT uptake in PLHIV |
|  | EMR | Computer generated eligibility reduces missing people |
| *Disadvantages CAT* | CAT results in a loss of autonomy | The routine character, guided questions and the reminding aspect of CAT gives HCW less autonomy on creating the best care cascade (including whether to prescribe TPT). This could be perceived as something negative. |
|  | Dissatisfied with care provided | HCW are not satisfied with how they provide care when using CAT |
|  | No room to think outside the box | The structured character leaves no room to think outside the box in terms of treatment |
|  | Mistrust CAT | This entails ideas as: “when we use CAT, we get lazy, and completely trust the system without checking” |
|  | CAT might lead to over-prescription of TPT | This not checking could lead to prescribing TPT to people who are actually not eligible |
| *How HCW think CAT works* | No added value nor improvement | HCWs don’t know what CAT adds to TPT prescription or how it could improve TPT prescription |
|  |  |  |
|  |  |  |
